# Meta-analysis and co-expression analysis revealed stable QTL and candidate genes conferring resistances to Fusarium and Gibberella ear rots while reducing mycotoxin contamination in maize

**DOI:** 10.3389/fpls.2022.1050891

**Published:** 2022-10-31

**Authors:** Félicien Akohoue, Thomas Miedaner

**Affiliations:** State Plant Breeding Institute, University of Hohenheim, Stuttgart, Germany

**Keywords:** Candidate genes, FUM and DON contaminations, Fusarium and Gibberella ear rots, genomic selection, QTL meta-analysis, type of resistance, *Zea mays* L.

## Abstract

Fusarium (FER) and Gibberella ear rots (GER) are the two most devastating diseases of maize (*Zea mays* L.) which reduce yield and affect grain quality worldwide, especially by contamination with mycotoxins. Genetic improvement of host resistance to effectively tackle FER and GER diseases requires the identification of stable quantitative trait loci (QTL) to facilitate the application of genomics-assisted breeding for improving selection efficiency in breeding programs. We applied improved meta-analysis algorithms to re-analyze 224 QTL identified in 15 studies based on dense genome-wide single nucleotide polymorphisms (SNP) in order to identify meta-QTL (MQTL) and colocalized genomic loci for fumonisin (FUM) and deoxynivalenol (DON) accumulation, silk (SR) and kernel (KR) resistances of both FER and GER, kernel dry-down rate (KDD) and husk coverage (HC). A high-resolution genetic consensus map with 36,243 loci was constructed and enabled the projection of 164 of the 224 collected QTL. Candidate genes (CG) mining was performed within the most refined MQTL, and identified CG were cross-validated using publicly available transcriptomic data of maize under *Fusarium graminearum* infection. The meta-analysis revealed 40 MQTL, of which 29 were associated each with 2-5 FER- and/or GER-related traits. Twenty-eight of the 40 MQTL were common to both FER and GER resistances and 19 MQTL were common to silk and kernel resistances. Fourteen most refined MQTL on chromosomes 1, 2, 3, 4, 7 and 9 harbored a total of 2,272 CG. Cross-validation identified 59 of these CG as responsive to FER and/or GER diseases. MQTL *ZmMQTL2.2*, *ZmMQTL9.2* and *ZmMQTL9.4* harbored promising resistance genes, of which *GRMZM2G011151* and *GRMZM2G093092* were specific to the resistant line for both diseases and encoded “*terpene synthase21 (tps21)*” and “*flavonoid O-methyltransferase2 (fomt2)*”, respectively. Our findings revealed stable refined MQTL harboring promising candidate genes for use in breeding programs for improving FER and GER resistances with reduced mycotoxin accumulation. These candidate genes can be transferred into elite cultivars by integrating refined MQTL into genomics-assisted backcross breeding strategies.

## Introduction

Maize (*Zea mays* L.) is the most important cereal crop in terms of grain production volume worldwide, and is set to become the first commercial crop in the coming decade (Shiferaw et al., 2011; Erenstein et al., 2022). The increase in production over the past quarter century was supported by more than 46 and 50% increase in area expansion and grain yield, respectively (Erenstein et al., 2022). Despite this remarkable progress and intensive research and development efforts deployed, maize production is still threatened by many biotic stress factors which are expected to worsen with the changing climate (Grote et al., 2021). About 38 pests and diseases were recently reported to cause 19–41% grain losses in maize on the global scale (Savary et al., 2019). Among these, Fusarium and Gibberella ear rots represent major yield- and quality-impacting maize diseases which occur across regions and countries (Eckard et al., 2011; Beukes et al., 2018; Ma et al., 2019; Perincherry et al., 2019; Machado et al., 2022).

Fusarium ear rot (FER) or “pink ear rot” is mainly caused by the *Fusarium fujikuroi* species complex with *F. verticillioides* (Sacc.) Nirenberg being the most harmful pathogen distributed across all continents with higher aggressiveness in warmer climatic regions (Boutigny et al., 2011; Tsehaye et al., 2017; Ncube et al., 2020). Meanwhile, Gibberella ear rot (GER), also known as “red ear rot” or “red fusariosis”, is one of the most important maize ear rots in cooler climate zones, which is associated with the *F. graminearum* species complex with *F. graminearum sensu strictu* Schwabe (teleomorph *Gibberella zeae*) as the most dominant causal agent reported in North America, Australia, China and Europe (Gromadzka et al., 2016; Beukes et al., 2018; Castañares et al., 2019; Crippin et al., 2020; Pfordt et al., 2020; Dalla Lana et al., 2021; Machado et al., 2022). With the global changing climate and local weather variability and cultivation systems, both FER and GER are also frequently found on maize ears in the same locations with varying degrees of severity (Scauflaire et al., 2011; Schjøth and Sundheim, 2013; Shala-Mayrhofer et al., 2013; Pfordt et al., 2020; Czarnecka et al., 2022). Depending on the Fusarium species, different types of harmful mycotoxins are produced, of which fumonisins (FUM) and deoxynivalenol (DON) are the most predominant for FER and GER, respectively. FER and GER significantly reduce maize production and the accumulated mycotoxins can make the grains toxic for human consumption and animal feeding (Battilani and Logrieco, 2014; Logrieco et al., 2021).

Disease management practices such as tillage, crop rotation and fungicide application have minor effects on FER and GER severity and do not significantly increase the grain yield (Andriolli et al., 2016; Scarpino et al., 2018; Pfordt et al., 2020). In addition, available mycotoxin reduction technologies are labor- and cost-prohibitive, leading to a low adoption by farmers (Logrieco et al., 2021). Effective management strategies of FER and GER diseases and associated mycotoxins should consider integrating not only improved and environmentally friendly practices, but also improving plant resistance to the pathogens.

Several studies have reported germplasms with different levels of resistance to FER and GER worldwide (Reid et al., 2001a; Reid et al., 2001b; Reid et al., 2003; Gaikpa et al., 2021; Galiano-Carneiro et al., 2021). In Europe, Gaikpa et al. (2021) evaluated two European flint landrace populations (“*Kemater Landmais Gelb*” and “*Petkuser Ferdinand Rot*”) and identified resistant lines which can be used for developing high-yielding hybrid cultivars with improved resistance to GER. In Canada, inbred lines with high resistance to FER and GER have been reported by Reid et al. (2001a; 2001b; 2003). Similarly, potential sources of resistance to FER were identified in China (Guo et al., 2020) and tropical regions including southern, western and central Africa (Tembo et al., 2022). The exploitation of existing resistance sources in breeding programs requires a clear understanding of the genetic architecture of FER- and GER-related traits, and underlying molecular mechanisms. FER and GER resistances are complex traits which were reported to be quantitatively inherited and are thus controlled by numerous quantitative trait loci (QTL) (Martin et al., 2012a; Butrón et al., 2015).

More than 300 QTL were reported for both FER and GER resistances and related traits in different mapping populations by applying both low-throughput technologies, namely single sequence repeats (SSR), restriction fragment length polymorphisms (RFLP) and random amplified polymorphic DNA (RAPD) (Ali et al., 2005; Robertson-Hoyt et al., 2006; Li et al., 2011; Martin et al., 2011; Martin et al., 2012b), and dense genome-wide high-throughput technologies such as single nucleotide polymorphisms (SNP) (Giomi et al., 2016; Han et al., 2016; Kebede et al., 2016; Han et al., 2018; Wen et al., 2020; Yuan et al., 2020a; Gaikpa et al., 2021; Galiano-Carneiro et al., 2021; Zhou et al., 2021). This impressive amount of QTL reported through diverse studies offers a possibility for the application of genomics-assisted breeding strategies to efficiently and accurately improve ear rot resistances in maize. However, due to the complex nature of the traits, the application of these loci in breeding programs remains challenging and limited. Therefore, in order to make reported QTL more useful and facilitate their successful incorporation into breeding programs, a comprehensive and in-depth analysis of these loci needs to be carried out using appropriate statistical approaches like meta-analysis. QTL meta-analysis is an efficient approach which was developed by Goffinet and Gerber (2000) and has constantly improved during the past decade (Salvi and Tuberosa, 2015). The analysis allows the compilation of QTL observed in independent studies which are projected onto a consensus map in order to verify whether they represent a common genomic region on the genetic map or whether they correspond to different loci (Venske et al., 2019). This approach enables the identification of more refined and stable “real” QTL, also referred to as meta-QTL (MQTL), which are mostly involved in the variation of the traits. Moreover, in resistance breeding, the application of meta-analysis would help to identify refined (i.e. smaller in length) genomic regions which confer multi-disease resistances in crops. Furthermore, refined MQTL facilitate the identification and validation of candidate genes that are effectively involved into the variation of the traits. QTL meta-analysis has been successfully implemented to depict genetic architecture of different traits including Fusarium head blight (FHB) resistance and abiotic stress traits in wheat (*Triticum aestivum* L.) (Venske et al., 2019; Soriano et al., 2021), maize streak disease and low temperature tolerance in maize (Emeraghi et al., 2021; Yu et al., 2022) and nitrogen use efficiency in rice (*Oryza sativa* L.) (Sandhu et al., 2021).

To date, three QTL meta-analyses based on SSR and RFLP markers have been conducted on ear rot diseases in maize (Xiang et al., 2010; Xiang et al., 2012; Mideros et al., 2014). These authors included only one GER-related study by Ali et al. (2005), while the others were on FER- and *Aspergillus flavus*-caused ear rots. Moreover, SSR, RFLP and RAPD are low-throughput and complicated marker technologies which are unable to precisely identify the number and locations of genes controlling the traits, thereby leading to large QTL intervals (Yu et al., 2011; Venske et al., 2019). In addition, the identified MQTL lacked precision on flanking markers and genomic positions to enable identification of promising candidate genes to be targeted in breeding programs. With this, these studies can be considered as preliminary and more informational QTL meta-analyses on ear rot diseases.

In the subsequent years after these studies, there has been a revolution concerning genotyping technologies which led to the development of high throughput technologies for SNP including maizeSNP50 and Affymetrix microarray CGMB56K (Ganal et al., 2011), maizeSNP3072 (Tian et al., 2015) and GenoBaits maize10K (Guo et al., 2019) SNP arrays, as well as genotype-by-sequencing (GBS) technology (He et al., 2014) which can assess thousands of SNP at once. This has enabled the implementation of various QTL mapping studies, resulting in the accumulation of relevant information on QTL for FER and GER resistances and related traits, which should be jointly re-analyzed and updated to inform maize breeding strategies.

This study aims to (i) re-analyze and refine quantitative trait loci (QTL) reported by independent SNP-based QTL mapping studies for FER and GER silk resistance, kernel resistance, fumonisins and deoxynivalenol accumulation, kernel dry-down rate and husk coverage by applying a meta-analysis approach for identifying refined MQTL with precise genomic positions, thus revealing colocalization of genomic regions among the traits; (ii) identify candidate genes and (iii) describe the molecular mechanisms underlying resistance/susceptibility to FER and GER by analyzing the transcriptomic profiles of two contrasting maize lines (resistant *vs.* susceptible). To effectively identify most refined and stable MQTL, only SNP-based QTL mapping studies were included in the meta-analysis.

## Materials and methods

### Search strategy

To address our research questions, a paper-wise search was performed following the procedure described by Venske et al. (2019) and the updated guideline for systematic reviews and meta-analysis by Page et al. (2021). Searches were implemented in SCOPUS web-based, Web of Science (WoS) and Google Scholar (GoS) databases. To optimize search output, we used a combination of search terms and Boolean operators as follows: “ear rot” AND QTL AND (maize OR corn). Searches were done within the title, abstract and authors’ keywords in SCOPUS and WoS, and within the title in GoS. Afterwards, the search results were firstly exported as Research Information System (RIS) and Comma-Separated Values (CSV) formats and merged to remove duplicates. Secondly, all unique publications were considered for a first screening based on the publication language, type, subject area, focus of the study, content, marker type and data availability (**Table 1**). Thirdly, publications that satisfied the inclusion criteria were further screened to collect relevant information about the reported QTL. For each QTL, key information was collected on: (i) traits; (ii) sources of resistance; (iii) type and size of the mapping populations; (iv) logarithm of odds (LOD) score; (v) proportion of phenotypic variance explained by the QTL as measured by R^2^; (vi) most closely flanking or single markers for interval mapping and single marker analysis, respectively; (vii) peak position and 95% confidence interval (CI) of the QTL (**Supplementary File 1**). LOD score was considered equal to 3 for single marker analysis where the exact LOD value was not reported. For studies which reported the genotypic variance explained (p_G_) by QTL, we estimated the corresponding phenotypic variance (PVE) as follows:


(1) $$PVE\;=p_{G}\;x\;H^{2}$$

**Table 1:** Inclusion and exclusion criteria.

Criteria	Inclusion
Publication language	English and/or French
Document type	Original research articles, books or book chapters
Subject	Agricultural and Biological Sciences
Focus	Fusarium ear rot (FER) in maize Gibberella ear rot (GER) in maize
Search string	“ear rot” AND QTL AND (maize OR corn)
Content	Mapping of quantitative trait loci (QTL) conferring resistance to Fusarium ear rot (FER) and Gibberella ear rot (GER) in maize.
Marker technology	Single nucleotide polymorphisms (SNP)
Data	Availability of sufficient information to enable proper meta-analysis of QTL associated with FER and/or GER

where H^2^ is the heritability reported for the trait by the respective study. QTL with PVE<10%, 10%≤PVE<20% and PVE≥20% was considered as having minor, medium and major-effect on the trait, respectively. From the QTL mapping studies, six FER- and GER-related traits were collected and included in our meta-analysis: fumonisin accumulation (FUM), deoxynivalenol accumulation (DON), husk coverage (HC), kernel dry-down rate (KDD), kernel resistance (KR) and silk resistance (SR). FUM and DON were specific to FER and GER, respectively.

### Consensus map construction

To project all the QTL collected from the diverse studies, a consensus map was constructed based on a linear programming algorithm in the LPmerge R package (Endelman and Plomion, 2014) which efficiently minimizes the error between markers’ positions on the consensus map and the individual linkage maps. Based on the sequencing technology used in the original studies, a total of eight high-quality genetic maps which harbored a large number of SNP markers were selected and included in the analysis. For chip-based SNP markers, high-resolution consensus maps were obtained from Ganal et al. (2011); Liu et al. (2015) and Wen et al. (2020) for Illumina maizeSNP50, IBM Syn10 and GenoBaits maize10K SNP arrays, respectively. For GBS technology, we included the genetic map from Kebede et al. (2016). In addition, four linkage maps used by Giomi et al. (2016); Chen et al. (2016); Maschietto et al. (2017) and Zhou et al. (2021) were also included in the analysis. In the procedure, markers were assigned to bins based on their co-segregation, and the maximum interval between bins was set to k = 1−3. Thus, one consensus map was produced for each k value. The best k and corresponding consensus map were selected based on the root-mean-squared error (RMSE) between the consensus map and the linkage maps. The lower the RMSE, the higher the resolution of the respective consensus map. Spearman rank correlation analysis was performed to evaluate the degree of preservation of marker order between the consensus map and the individual genetic maps as well as the collinearity between the consensus map and the physical map B73 RefGen_v2. The proportion of markers which were arranged in the same order with those on the corresponding chromosomes on the physical map was also estimated. All analyses were conducted using R software v4.1.0 (R Core Team, 2021).

### Meta-analysis of quantitative trait loci

QTL were projected onto the consensus map previously developed to identify MQTL on each linkage group. All projected QTL had their flanking markers information on at least one of the individual maps used to generate the consensus map. Prior to the projection, the confidence interval (CI) at 95% was estimated for each QTL using the following empirical formula described for each mapping population by Darvasi and Soller (1997) and Guo et al. (2006):


(2) $${\text{F}}_{2}:\text{CI = }\frac{530}{{\text{N x R}}^{2}}$$

(3) $$\text{Double haploid }\left(\text{DH}\right):\text{CI = }\frac{287}{{\text{N x R}}^{2}}$$

(4) $$\text{Recombinant inbred lines }\left(\text{RIL}\right):\text{CI = }\frac{163}{{\text{N x R}}^{2}}$$

where N is the number of lines and R^2^ is the phenotypic variance explained by the QTL.

Afterwards, the calculated confidence intervals, original LOD score, R^2^, QTL most likely position (middle point), as well as start and end positions (**Supplementary File 1**), were projected onto the consensus map using the *Veyrieras* two-step clustering procedure based on a Gaussian mixture model which parameter estimates were obtained by applying the expectation-maximization (EM) algorithm in BioMercator V4.2.3 software (Arcade et al., 2004; Veyrieras et al., 2007; Sosnowski et al., 2012). Considering the known correlations among the traits, the QTL were analyzed together as one trait referred to as DT (Chungu et al., 1996; Löffler et al., 2010a; Kebebe et al., 2015; Kebede et al., 2016). In the first step (1/2), the projected QTL were clustered on each chromosome or linkage group assuming varying numbers of MQTL or “real QTL” (k). The maximum number of MQTL (kmax) was the total number of QTL on the linkage group minus one QTL. For example, on a linkage group with 20 QTL, kmax was set to 19. The number of random starting points and convergence threshold for the EM algorithm were set to 50 and 1.e^-8^, respectively. MQTL model with the best k was the one showing the lowest value and the highest weight for at least three of the following parameters: Akaike Information Criterion (AIC), corrected Akaike Information Criterion (AICc and AIC_3_), Bayesian Information Criterion (BIC) and Average Weight of Evidence (AWE). In the second step (2/2), the k MQTL were displayed according to the chosen model (Veyrieras et al., 2007). Each MQTL was represented by at least two original QTL with overlapping confidence intervals, and shared no QTL with other MQTL on the same chromosome (Yu et al., 2022). With this, original QTL which overlapped with two or more MQTL were discarded from the analysis. The position of the MQTL was determined based on the mean of the original QTL distribution maximizing the likelihood. The phenotypic variance explained by each MQTL was calculated as the mean R^2^ of the original respective QTL (Yu et al., 2022). Furthermore, the meta-analysis was compared with marker-trait associations (MTA) studies by identifying the number of MTA reported for each trait, which were located within identified MQTL.

### Candidate genes mining and expression analysis

From the meta-analysis, we selected the most refined MQTL which were considered for candidate genes (CG) mining and transcriptomic analysis. MQTL were selected using the criteria described by Venske et al. (2019) and Soriano et al. (2021) as follows: (1) the selected MQTL was constituted by at least two overlapping original QTL; (2) CI (95%) of the MQTL was lower than the average CI of the respective QTL; (3) MQTL was shorter than 20 Mbp in physical distance; (4) and phenotypic variance explained by the MQTL was equal or greater than 10%. Candidate genes within each of the selected MQTL were mined based on the physical positions of flanking markers by surveying the maize annotation browser of the reference genome (B73 RefGen_v3) which is available from the MaizeGDB database (Lawrence, 2007) (https://www.maizegdb.org/gbrowse/maize_v3). Physical positions of flanking markers were obtained from Unterseer et al. (2016); Kebede et al. (2016) and Liu et al. (2015). Low confidence genes and transposable elements were excluded.

To identify which of these CG were differentially expressed when challenged with *F. graminearum*, we conducted a transcriptional expression analysis based on RNA-Seq data for Gibberella ear rot published by Kebede et al. (2018) available from the NCBI Gene Expression Omnibus (GSE92448) (https://www.ncbi.nlm.nih.gov/geo/query/acc.cgi?acc=GSE92448). The authors evaluated over two years (2004 and 2006) the transcriptomic profiles of two maize lines; CO441 (FER and GER resistant) and B37 (FER and GER susceptible) under control conditions (mock) and after inoculation with *F. graminearum*. Inoculation was done 11 days after controlled pollination using the kernel inoculation method (Reid et al., 2002; Kebede et al., 2018). Maize ears were collected one and two days after inoculation (DAI) and RNA was extracted in bulk per testing year from developing kernels (Kebede et al., 2018). Gene expression levels were determined based on mock vs. Fusarium comparisons by calculating transcripts per million (TPM) as follows:


(5) $$TPM=\;\frac{RPKM_{i}\times 10^{6}}{\sum_{i}^{n}RPKM}$$

where *RPKM_i_* is the reads per kilobase million of the *i^th^* gene/transcript, and *n* is the total number of genes/transcripts. RPKM was estimated for each gene based on the total exon reads (ER), mapped reads (MR, in millions) and exon length (EL, in kb) as:


(6) $$RPKM=\frac{ER}{MR\times EL}$$

According to Kebede et al. (2018), genes were considered as differentially expressed if the respective corrected False discovery rate (FDR) p-value was equal or lower than 0.05, fold change≥2 and TPM≥5. The differentially expressed genes identified through the transcriptomic analysis where further searched for protein evidence against the MaizeGDB (Lawrence, 2007) and the Nation Center for Biotechnology Information (NCBI, https://www.ncbi.nlm.nih.gov/) to identify corresponding annotations and ontology terms.

## Results

### Identification and screening of relevant publications for FER- and GER-related traits

Based on the search terms indicated previously, a total of 153 papers were identified from SCOPUS (64), WoS (55) and GoS (34) as described by the preferred reporting items for systematic review and meta-analyses (PRISMA) flow diagram available in **Supplementary File 2**. From this, 43 unique publications were obtained with publication year ranging from 1993 to 2022 after removing duplicates (89), review articles and meta-analyses (11) and publications related to trait inheritance (1), gene expression (8) and FER resistance on seedlings (1). One paper published in Chinese was removed (Wen et al., 2021a). Five (9.3%) publications were solely focused on Aspergillus ear rot (Busboom and White, 2004; Willcox et al., 2013; Smith et al., 2019) and one publication on Diplodia ear rot (Baer et al., 2021), and were therefore excluded. This resulted into 37 papers which focused on deciphering the genetic architecture of FER- and GER-related traits in maize. Fifteen of these papers concentrated on QTL identification based on low-throughput technologies such as SSR, RFLP, and RADP markers (Pè et al., 1993; Ali et al., 2005; Martin et al., 2011; Martin et al., 2012b), and validation of QTL reported in previous studies (Martin et al., 2012c; Brauner et al., 2017). In addition, one SNP-based QTL mapping publication was excluded due to missing information on QTL genetic position, flanking markers as well as LOD score and PVE (Morales et al., 2019). Finally, 22 publications satisfied our inclusion criteria and were therefore considered for full text screening. Fifteen publications were SNP-based QTL mapping studies which were used to collect relevant information required for the QTL meta-analysis (**Supplementary File 2**) (Chen et al., 2016; Giomi et al., 2016; Han et al., 2016; Kebede et al., 2016; Maschietto et al., 2017; Han et al., 2018; Galić et al., 2019; Wen et al., 2020; Yuan et al., 2020a; Galiano-Carneiro et al., 2021; Giomi et al., 2021; Wen et al., 2021b; Zhou et al., 2021; Feng et al., 2022; Guo et al., 2022). Seven papers were related to genome-wide association study and used to cross-validate the meta-analysis (Butrón et al., 2019; Samayoa et al., 2019; Wu et al., 2020; Gaikpa et al., 2021; Gesteiro et al., 2021; Liu et al., 2021; da Silva et al., 2022).

### Characterization of QTL reported based on high-throughput SNP technologies for FER- and GER-related traits

From the 15 SNP-based QTL mapping studies, a total of 224 QTL were reported for FER- and GER- related traits (**Table 2**, **Supplementary File 1**). QTL were identified using three types of populations such as recombinant inbred lines (RIL), double-haploid (DH) and F_2_ populations. Resistant parental lines used in the different studies were sourced from a wide distribution range including Argentina, Brazil, Canada, China, Europe, United States of America (USA), and the International Maize and Wheat Improvement Center (CIMMYT).

**Table 2:** Characteristics of SNP-based QTL mapping studies on resistance to Fusarium (FER) and Gibberella ear rot (GER) analysed in this study.

Donor	Origin	Type	Size	Disease	Traits	Number of QTL	References
LP4637	Argentina	RIL	298	GER	SR	8	Giomi et al., 2016
CO441	Canada	RIL	410	GER	SR, KR, KDD, HC	32	Kebede et al., 2016
European flint	Europe	DH	114	GER	DON	6	Han et al., 2018
European dent	Europe	DH	130	GER	DON	2	Han et al., 2018
Cheng351	China	F2	118	GER	SR	3	Wen et al., 2020
Dan598	China	F2	200	GER	SR	8	Wen et al., 2020
JiV203	China	F2	175	GER	SR	11	Wen et al., 2020
IBMSyn10	USA	DH	298	GER	SR	1	Yuan et al., 2020a
DH4866	China	RIL	204	GER	KR	11	Zhou et al., 2021
T3	Brazil	DH	266	GER	SR	3	Galiano-Carneiro et al., 2021
UH006 and UH007	Europe	DH	639	GER	SR, DON	22	Han et al., 2016
CML495	CIMMYT	DH	201	FER	KR	4	Chen et al., 2016
CML449	CIMMYT	F2	272	FER	KR	6	Chen et al., 2016
CML492	CIMMYT	F2	277	FER	KR	11	Chen et al., 2016
CO441	Canada	F2	188	FER	FUM, KR	24	Maschietto et al., 2017
IBMSyn4	USA	RIL	191	FER	KR	3	Galić et al., 2019
LP4637	Argentina	RIL	120	FER	SR	7	Giomi et al., 2021
Cheng351	China	F2	117	FER	KR	5	Wen et al., 2021b
Dan598	China	F2	200	FER	KR	10	Wen et al., 2021b
JiV203	China	F2	174	FER	KR	15	Wen et al., 2021b
DTMA165	CIMMYT	F2	152	FER	KR	9	Guo et al., 2022
8107	China	F2	220	FER	KR	8	Guo et al., 2022
B73xdiploperennis	China	RIL	215	FER	KR	7	Feng et al., 2022
B73xparviglumis	China	RIL	113	FER	KR	3	Feng et al., 2022
Zheng58xparviglumis	China	RIL	122	FER	KR	5	Feng et al., 2022

CIMMYT, International Maize and Wheat Improvement Center; RIL, recombinant inbred lines; DH, double haploid; DON, deoxynivalenol accumulation; FUM, fumonisin accumulation; HC, husk coverage; KDD, kernel dry-down rate; KR, kernel resistance; SR, silk resistance.

**Figure 1:**
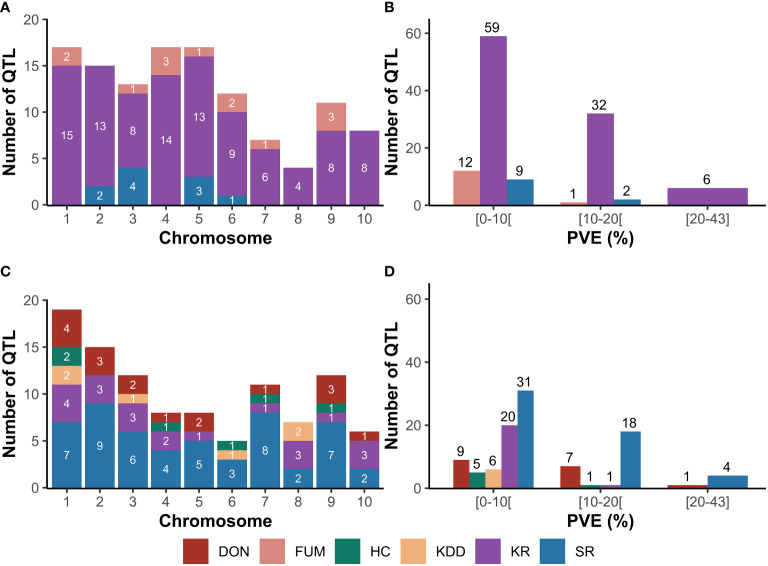
Original QTL reported from SNP-based mapping studies for Fusarium ear rot (FER) and Gibberella ear rot (GER). **(A)**, distribution of QTL for FER across chromosomes; **(B)**, phenotypic variance explained (PVE) by QTL for FER; **(C)**, distribution of QTL for GER across chromosomes; **(D)**, phenotypic variance explained by QTL for GER. DON, deoxynivalenol accumulation; FUM , fumonisin accumulation; HC, husk coverage; KDD, kernel dry-down rate; KR, kernel resistance; SR, silk resistance.

Considering the three FER-related traits, 121 QTL were reported and distributed across all chromosomes (**Figure 1A**). Thirteen QTL were reported for FUM on all chromosomes except for chromosomes 2, 8 and 10, while 97 QTL were identified for KR on all chromosomes. Eleven QTL were identified for SR across chromosomes 2, 3, 5, and 6 (**Figure 1A**). Twelve and one QTL for FUM exhibited minor (PVE<10%) and medium (10%≤PVE<20%) effects, respectively (**Figure 1B**). 32 and six QTL for KR had medium and major effects (PVE≥20%), respectively. In addition, nine and one QTL for SR exerted minor and medium effects on the trait, respectively (**Figure 1B**).

For the five GER-related traits, 103 QTL were identified across all chromosomes (**Figure 1C**). A total of 17 QTL were reported for DON on all chromosomes except for chromosomes 6 and 8, while 21 QTL were identified for KR on all chromosomes except chromosome 6. 53 QTL were reported for SR across all chromosomes. Six QTL were identified for HC across chromosomes 1, 4, 6, 7 and 9, while six QTL were reported for KDD on chromosomes 1, 3, 6 and 8 (**Figure 1C**). Seven and one QTL for DON had medium and major effects, respectively, while most QTL for KR (20 QTL) exhibited minor effects (**Figure 1D**). Similarly, 18 and four of the 53 QTL for SR had medium and major effects, respectively. Most QTL for HC (5 QTL) and all QTL for KDD had minor and medium effects on the traits (**Figure 1D**).

### High-resolution consensus map generated for QTL projection

The consensus map was composed of SNP markers and generated based on eight genetic linkage maps. The map was of high resolution and presented a total of 36,243 loci with a total length of 3,132.48 cM (**Table 3**). The Spearman rank correlation analysis revealed strong correlations (average ρ = 0.86−0.99) between marker order on the consensus and individual genetic maps (**Table 3**). Each chromosome was, on average, 313.25 cM long and composed of 3,624 SNP markers. The average genetic distance between adjacent markers ranged from 0.15 to 0.28 cM depending on the chromosome (**Table 3**). Attempts to increase the number of loci and length of the map through the inclusion of additional genetic maps resulted in several conflict orders. A comparison of the consensus map with physical map obtained from the reference map B73 RefGen_v2, showed high collinearity with strong correlations (ρ = 0.73−0.91). On average, 72% of markers were arranged in the same order with those on the corresponding chromosomes of the physical map, indicating a high consistency between the consensus map and the physical map B73 RefGen_v2. This shows that the current consensus map generated in this study was the best harmonious combination, and was therefore used as the base for the QTL projection and meta-analysis. The consensus map is made available through **Supplementary File 3**.

**Table 3:** Characteristics of consensus map generated from eight high quality genetic maps composed of SNP markers.

Chr	Length (cM)	Number of markers	Average DM (cM)	Average ρ with IGM	Range of ρ with IGM	ρ with physical map	Consistent proportion (%)
1	450.72	5,839	0.20	0.88	0.82−0.95	0.80	0.68
2	316.00	4,001	0.28	0.97	0.91−1.00	0.86	0.71
3	463.30	4,074	0.28	0.98	0.96−0.99	0.85	0.70
4	319.29	3,876	0.24	0.99	0.99−0.99	0.87	0.73
5	318.31	3,885	0.27	0.95	0.84−1.00	0.75	0.73
6	120.26	3,093	0.15	0.86	0.77−0.99	0.73	0.69
7	371.10	3,175	0.24	0.80	0.61−0.95	0.74	0.71
8	287.80	3,059	0.20	0.95	0.88−1.00	0.80	0.72
9	254.90	2,696	0.25	0.98	0.96−1.00	0.91	0.71
10	230.80	2,545	0.26	0.98	0.97−1.00	0.83	0.72
Genome	3,132.48	36,243	0.24	0.93		0.81	0.72

Chr, chromosome; DM, distance between markers; ρ, Spearman rank correlation coefficient; IGM, individual genetic maps used for the consensus map construction. Physical map was obtained from the reference map B73 RefGen_v2. Consistent proportion is the proportion of markers arranged in the same order with those on the corresponding chromosomes of the physical map.

### QTL colocalization and meta-QTL for the FER- and GER-related traits based on QTL mapping studies

**Figure 2:**
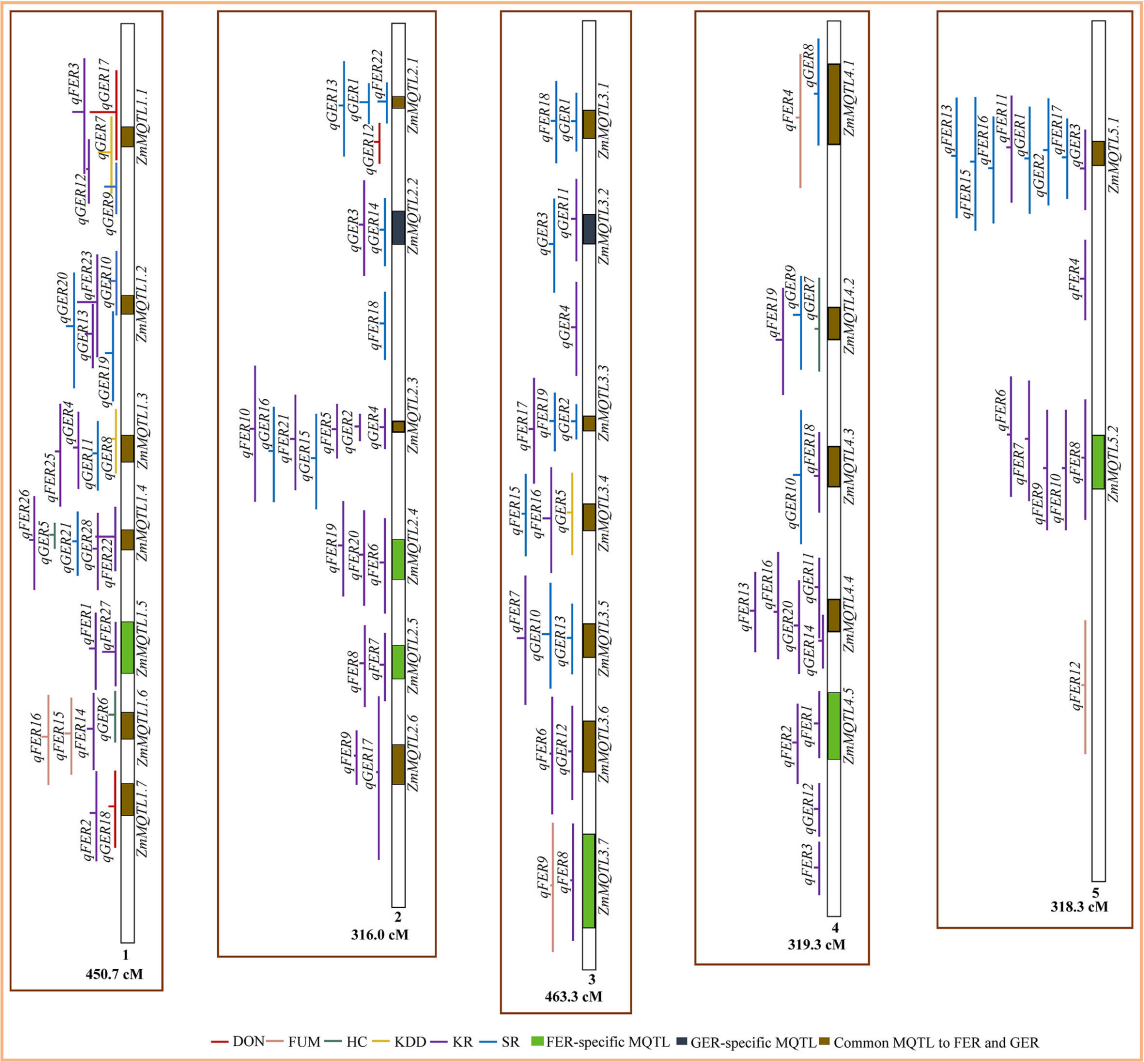
Colocalization of QTL for Fusarium ear rot (FER) and Gibberella ear rot (GER) and identification of meta-QTL (MQTL) on chromosomes 1‒5. The line in the middle of each QTL represents its LOD score in the original work. The longer this line, the higher the LOD score of the respective QTL. DON, deoxynivalenol accumulation; FUM, fumonisin accumulation; HC, husk coverage; KDD, kernel dry-down rate; KR, kernel resistance; SR, silk resistance.

**Figure 3:**
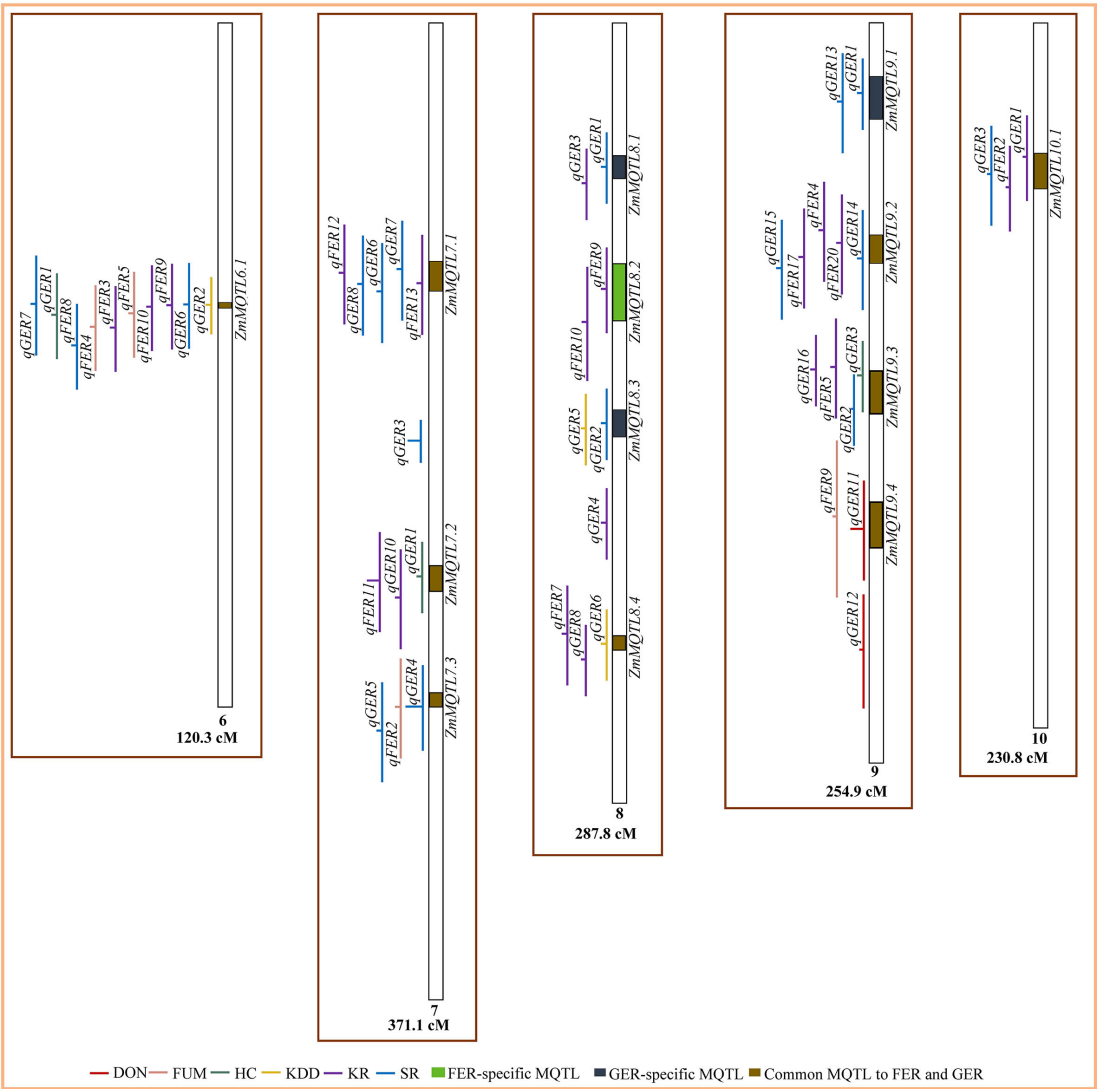
Colocalization of QTL for Fusarium ear rot (FER) and Gibberella ear rot (GER) and identification of meta-QTL (MQTL) on chromosomes 6‒10. The line in the middle of each QTL represents its LOD score in the original work. The longer this line, the higher the LOD score of the respective QTL. DON, deoxynivalenol accumulation; FUM, fumonisin accumulation; HC, husk coverage; KDD, kernel dry-down rate; KR, kernel resistance; SR, silk resistance.

From the total of 224 QTL, 164 QTL were projected on the consensus map (**Figures 2**, **3**). The remaining 60 QTL could not be projected due to lack of information (markers’ names and positions) on the flanking markers in the original studies (25 QTL) or the absence of the markers on the consensus map (35 QTL) generated in this study. For both FER and GER, the projection showed that confidence intervals of QTL for different traits overlapped on several chromosomes, indicating colocalization of resistance QTL for the two diseases with two or more traits. To refine MQTL, QTL with large confidence intervals (CI 95%≥80 cM) on chromosomes 1, 6 and 10 were excluded from the meta-analysis. Likewise, QTL which overlapped two or more independent MQTL on chromosomes 2, 4, 5, 7 and 9 were also excluded from the analysis. A total of 40 MQTL were identified across all chromosomes and constituted each by 2−10 overlapping original QTL (**Supplementary File 4**). On average, 70−100% of CI of individual QTL contributed to the definition of each MQTL. CI of identified MQTL were 1.4−36.4-fold lower than the average CI of respective original QTL. 32 of the 40 MQTL were constituted of original QTL from 2−7 different studies and populations (**Supplementary File 4**). The highest number of MQTL was observed on chromosomes 1 and 3 (**Figure 2**), and the lowest on chromosomes 6 and 10 (**Figure 3**). From the 40 MQTL, seven and five MQTL were specific to FER and GER, respectively, while 28 MQTL were common to both diseases.

Four and six MQTL were found for DON and FUM, respectively, while KR and SR of FER were controlled by 30 and 6 MQTL, respectively (**Supplementary File 4**). Sixteen and 24 MQTL were found for KR and SR of GER, respectively, while HC and KDD were controlled by six MQTL each (**Supplementary File 4**). Contrary to KR and SR, no specific MQTL where identified for FUM, DON, HC and KDD. However, the analysis identified individual QTL *qFER12* on chromosome 5 and *qGER12* on chromosome 9 as independent specific QTL for FUM and DON, respectively. Considering both diseases, several MQTL were shared among the traits, with the exception of DON versus HC (**Table 4**). Four MQTL were shared between KR and SR of FER, while 15 MQTL were common to KR of FER and SR of GER (**Table 4**).

**Table 4:** Number of meta-QTL shared among the evaluated traits.

Trait	Fusarium ear rot			Gibberella ear rot				
	FUM	KR	SR	DON	HC	KDD	KR	SR
**Fusarium ear rot:**								
KR	3	‒						
SR	1	4	‒					
**Gibberella ear rot:**								
DON	1	2	1	‒				
HC	2	6	1	0	‒			
KDD	1	5	2	1	1	‒		
KR	0	13	1	1	3	3	‒	
SR	3	15	5	2	4	4	11	‒

DON, Deoxynivalenol accumulation; FUM, fumonisin accumulation; HC, husk coverage; KDD, kernel dry-down rate; KR, kernel resistance; SR, silk resistance. Each meta-QTL was common to different pairs of traits.

### Comparison of meta-analysis with association mapping studies

Based on the seven association mapping studies on FER and GER resistances, about 178 MTA were reported for FUM, KR of FER and SR of GER using diverse germplasm collections and breeding populations worldwide (**Table 5**). 170 MTA were reported for FER-related traits such as FUM (81 MTA) and KR (89 MTA). Depending on the traits, FER-related MTA were distributed across all chromosomes (**Supplementary File 5**). The remaining eight MTA were exclusively reported by one GER-related study (Gaikpa et al., 2021) for SR across chromosomes 2, 4, 5, 6, and 9 (**Supplementary File 5**). Unlike QTL, a single MTA does not have confidence interval, and was therefore considered as a specific QTL location, but not as a whole QTL. A cross-validation with the meta-analysis showed that physical positions of 33 of the reported MTA were located within 16 MQTL (**Table 6**). The proportion of MTA located within MQTL ranged from 7.14% on chromosome 2 to 50% on chromosome 8. No MTA reported on chromosomes 5, 6 and 10 fell within our MQTL (**Table 6**).

**Table 5:** Characteristics of association mapping studies on resistance to Fusarium (FER) and Gibberella ear rot (GER) used in this study for validation.

Donor	Type	Size	Disease	Trait	Number of MTA	References
Worldwide panel	IL	270	FER	FUM	38	Samayoa et al., 2019
EPS21 MAGIC population	RIL	352	FER	KR	13	Butrón et al., 2019
BT-1	RIL	250	FER	KR	18	Wu et al., 2020
Kemater Landmais Gelb landrace	DH	250	GER	SR	8	Gaikpa et al., 2021
CMLs, DTMA AM panel and SYN_DH	IL	874	FER	KR	58	Liu et al., 2021
EPS21 MAGIC population	RIL	339	FER	FUM	24	Gesteiro et al., 2021
Embrapa’s panel	IL	205	FER	FUM	19	da Silva et al., 2022

RIL, recombinant inbred lines; IL, inbred lines; DH, double haploid; FUM, fumonisin accumulation; KR, kernel resistance; SR, silk resistance.

**Table 6:** Number of marker-trait associations (MTA) located within identified meta-QTL (MQTL).

MQTL^a^	Physical position (Start−End, Mbp)	Trait and number of MTA^b^		MTA proportion (%)^c^	Source of resistant alleles	References
		FER	GER			
*ZmMQTL1.1*	7.09−9.68	KR (2)		22.72	Tropical maize germplasm, heterotic Tangsipingtou and Reid	Wu et al., 2020; Liu et al., 2021
*ZmMQTL1.5*	243.46−259.01	KR (1)			Tropical maize germplasm	Samayoa et al., 2019; Liu et al., 2021
*ZmMQTL1.6*	280.22−287.9	KR (2)			Tropical maize germplasm	Liu et al., 2021
*ZmMQTL2.2*	13.30−20.58		SR (1)	7.14	Kemater Landmais Gelb	Gaikpa et al., 2021
*ZmMQTL3.3*	164.70−168.68	KR (1)		20.00	Tropical maize germplasm	Liu et al., 2021
*ZmMQTL3.6*	211.85−215.42	KR (1)			EPS21 MAGIC population	Butrón et al., 2019
*ZmMQTL3.7*	219.19−229.39	KR (4)			Tropical maize germplasm	Liu et al., 2021
*ZmMQTL4.1*	2.10−5.24	FUM (2)		17.24	Worldwide panel	Samayoa et al., 2019
*ZmMQTL4.4*	173.55−180.3	KR (2)			EPS21 MAGIC population, CMLs, DTMA AM panel and SYN_DH	Butrón et al., 2019; Liu et al., 2021
*ZmMQTL7.1*	17.98−27.83	KR (1)		20.00	EPS21 MAGIC population	Butrón et al., 2019
*ZmMQTL7.2*	137.54−143.29	KR (1)			Tropical maize germplasm	Liu et al., 2021
*ZmMQTL7.3*	159.73−160.48	FUM (3)			EPS21 MAGIC population	Gesteiro et al., 2021
*ZmMQTL8.1*	4.11−12.94	KR (1)		50.00	EPS21 MAGIC population	Butrón et al., 2019
*ZmMQTL8.2*	20.80−81.7	KR (2)			Tropical maize germplasm	Liu et al., 2021
*ZmMQTL9.2*	113.95−129.03	KR (4)		44.44	Tropical maize germplasm	Liu et al., 2021
*ZmMQTL9.3*	137.29−141.47	KR (4)	SR (1)		Kemater Landmais Gelb, Tropical maize germplasm	Gaikpa et al., 2021; Liu et al., 2021

CI, confidence interval; FER, Fusarium ear rot; GER, Gibberella ear rot; FUM, fumonisin accumulation; KR, kernel resistance; SR, silk resistance.

a Meta-QTL name referred to Zea mays abbreviated as Zm, followed by MQTL, the corresponding chromosome, and identification number on the chromosome.

b Values in parentheses are the number of MTA for each trait, which are located within corresponding MQTL.

c Proportion of reported MTA per chromosome, which were located within MQTL.

### Differentially expressed candidate genes within the most refined MQTL

From the 40 MQTL identified in this study, 14 MQTL satisfied the four criteria defined earlier, and were therefore selected as the most refined MQTL (**Table 7**). Selected MQTL were distributed across chromosomes 1, 2, 3, 4, 7 and 9, with 2−7 overlapping original QTL. The CI was 2.65−14.80 cM, with an average PVE of 10−29.67%. The distance between flanking markers of the respective MQTL was 0.63−15.55 Mbp. Based on the physical positions of the flanking markers, a total of 2,272 candidate genes, excluding transposable elements, were mined within the confidence intervals of the selected MQTL (**Table 7**, **Supplementary File 6**). For each MQTL, an average of 162 CG were identified with the only exception of *ZmMQTL1.2*, where only 10 CG were projected. The highest number of CG was observed with *ZmMQTL4.3* (342 CG, **Table 7**).

**Table 7:** Selected meta-QTL (MQTL) and corresponding candidate genes (CG).

MQTL^a^	Number of QTL	Disease and trait		Number of Populations	PVE (%)	CI 95% (cM)	Physical distance (Mbp)	Number of CG
		**FER**	**GER**					
*ZmMQTL1.2*	5	KR	KR, SR	4	10.60	4.72	3.04	10
*ZmMQTL1.4*	5	KR	HC, KR, SR	5	14.00	5.85	7.00	146
*ZmMQTL1.5*	2	KR		2	11.50	14.80	15.55	331
*ZmMQTL1.7*	2	KR	DON	2	11.00	8.00	7.28	226
*ZmMQTL2.1*	4	SR	DON, SR	3	11.75	3.02	0.63	30
*ZmMQTL2.2*	2		KR, SR	2	13.00	9.74	7.28	201
*ZmMQTL2.3*	7	KR	KR, SR	5	10.00	2.65	6.18	68
*ZmMQTL3.3*	3	KR, SR	SR	2	10.00	3.75	3.98	77
*ZmMQTL4.3*	2	KR	SR	2	17.00	11.51	14.50	342
*ZmMQTL4.4*	5	KR	KR	2	13.40	8.89	6.75	155
*ZmMQTL7.1*	5	KR	SR	2	15.20	7.75	9.85	143
*ZmMQTL7.3*	3	FUM	SR	2	29.67	3.89	0.75	37
*ZmMQTL9.2*	5	KR	SR	3	10.40	8.00	15.08	304
*ZmMQTL9.4*	2	FUM	DON	2	13.50	11.71	5.94	202

CI, confidence interval; FER, Fusarium ear rot; GER, Gibberella ear rot; SR, silk resistance; KR, kernel resistance; DON, deoxynivalenol accumulation; FUM, fumonisin accumulation; KDD, kernel dry-down rate; HC, husk coverage; PVE, phenotypic variance explained.

a Meta-QTL name referred to Zea mays abbreviated as Zm, followed by MQTL, the corresponding chromosome, and identification number on the chromosome.

**Figure 4:**
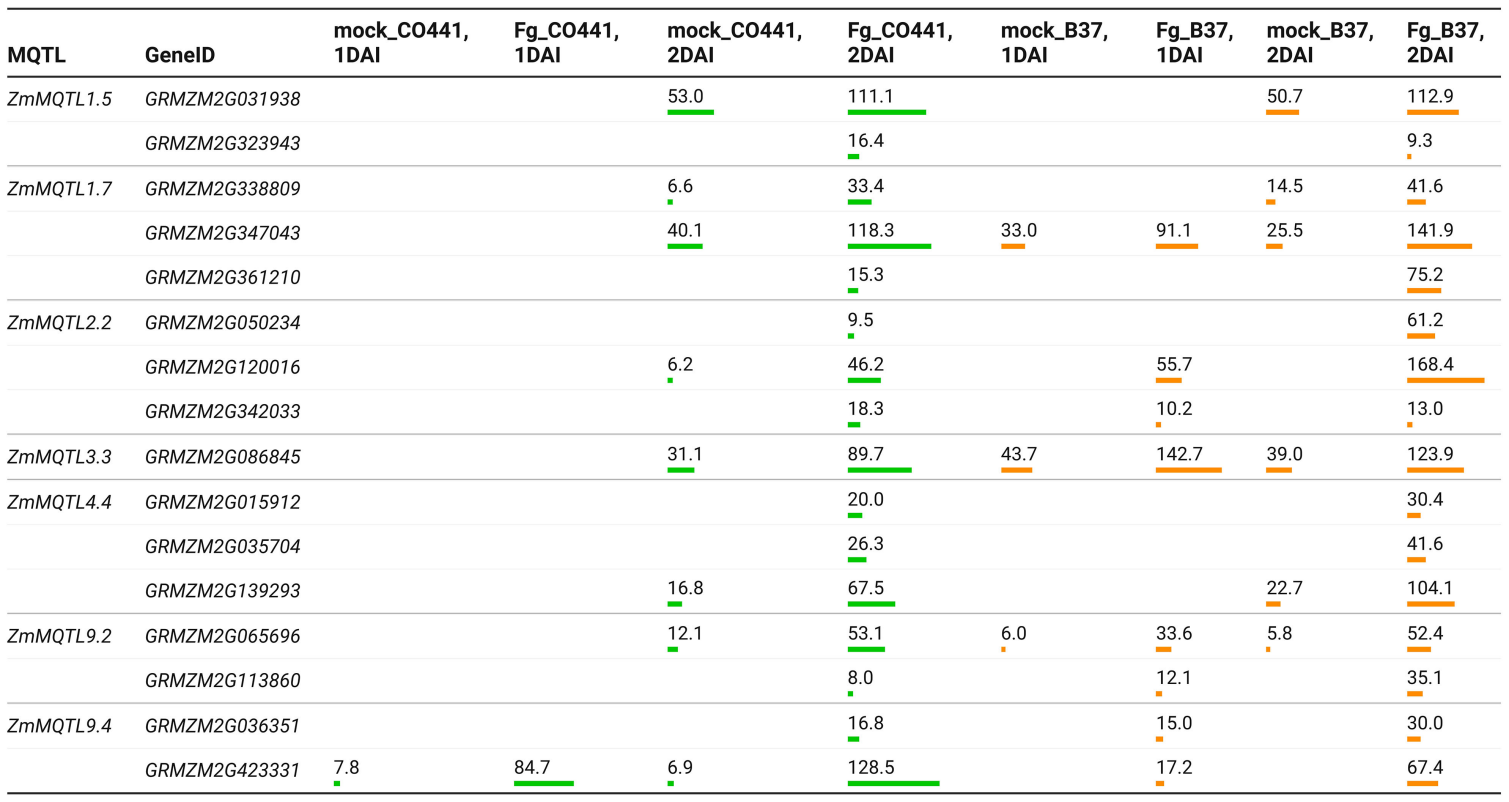
Expression levels in transcripts per million (TPM) of the common candidate genes in resistant (CO441) and susceptible (B37) lines under control conditions (mock) vs. *F. graminearum* (Fg) comparisons. Bar charts show the relative importance of the expression levels of each gene. MQTL, meta-QTL; DAI, days after inoculation.

Gene expression analysis using RNA-Seq data from Kebede et al. (2018), revealed that 59 of the CG were differentially expressed based on mock vs. Fusarium comparisons at 1−2 DAI (**Supplementary File 7**). Seven CG were specific to the resistant line (CO441), 36 to the susceptible line (B37) and 16 common to both lines. At 1 DAI, only genes *GRMZM2G093092* and *GRMZM2G423331* were differentially expressed in CO441, while 15 genes were differentially expressed in B37 (**Supplementary File 7**). Comparing to the respective controls (mock), all CG were upregulated in both lines, with the exception of *GRMZM2G135617*, *GRMZM2G164340* and *GRMZM2G126732*, which were specifically downregulated (Fold change = −3.3 to −5.7) in B37 at 2 DAI. Expression levels of line-specific genes were 19.6−387.6 TPM in CO441 and 4.6−481.9 TPM in B37 (**Supplementary File 7**). For the common CG, the expression levels were 6.2−128.5 TPM in CO441 and 6.0−168.4 TPM in B37 (**Figure 4**). At 2 DAI, the expression of common CG *GRMZM2G342033*, *GRMZM2G323943*, *GRMZM2G423331* were 1.5−2-fold higher in CO441 than B37.

**Figure 5:**
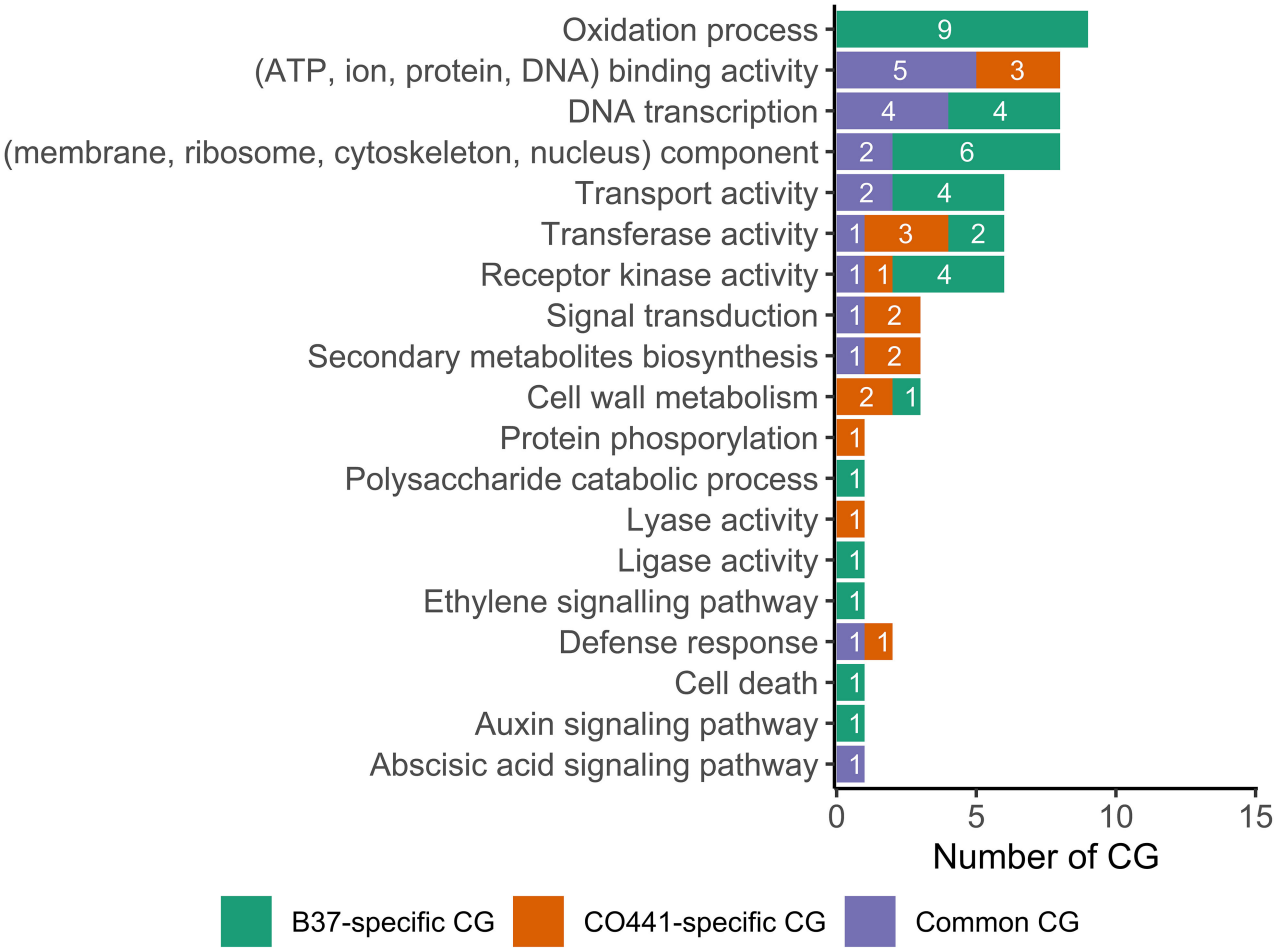
Gene ontology terms of the differentially expressed candidate genes (CG) between resistant (CO441) and susceptible (B37) lines under *Fusarium graminearum* infection.

Functional categories of 46 of the 59 differentially expressed CG were summarized in **Figure 5**. The remaining 13 CG, of which seven B37-specific CG, two CO441-specific CG (*GRMZM2G337191* and *GRMZM2G703858*) and four common CG, were annotated as “*uncharacterized protein*” (**Supplementary File 8**). Annotated CO441-specific CG were *GRMZM2G011151*, *GRMZM2G093092*, *GRMZM2G156785*, *GRMZM2G340656* and *GRMZM2G472643*, which were mainly involved in binding, kinase and transferase activities, signal transduction, secondary metabolism, cell wall metabolism and defense response (**Figure 5**, **Supplementary File 8**). Regarding the most important common CG (mostly expressed in CO441), *GRMZM2G342033* encoded “*S-norcoclaurine synthase2*” which was involved in lyase activity and defense response (**Supplementary File 8**). In addition, *GRMZM2G423331* encoded “*flavonoid O-methyltransferase4 (fomt4)*” which catalyzed sakuranetin (phytoalexin) biosynthesis and cell wall metabolism. Contrary to CO441-specific CG, no B37-specific CG was involved in defense response, signal transduction and secondary metabolites biosynthesis (**Figure 5**). Ethylene biosynthesis were catalyzed by “*1-aminocyclopropane-1-carboxylate synthase2 (acs2)*” encoded by *GRMZM2G164405*. Similarly, *GRMZM2G146108* encoded “*small auxin up RNA11 (saur11)*” which was involved in auxin biosynthesis. However, this gene was only highly expressed at 1 DAI. In addition, *GRMZM2G067402* encoded “*hemoglobin1 (hb1)*” which was involved in cell death under infection. Other B37-specific CG encoded many proteins which were involved in unspecific activities like ATP, ion and pyridoxal binding, oxidation-reduction process, transport and kinase activity (**Figure 5**, **Supplementary File 8**).

## Discussion

Based on dense genome-wide SNP technology, 224 QTL, of which 121 and 103 QTL for FER- and GER-related traits, respectively, have been reported during the last two decades in maize. These loci were jointly re-analyzed and clustered into a total of 40 more refined MQTL controlling one or more traits like DON, FUM, HC, KDD, KR and SR. Contrary to meta-analyses by Xiang et al. (2010); Xiang et al. (2012) and Mideros et al. (2014) based on low-throughput markers (RFLP, SSR and RAPD), and which included only one GER-related study, the MQTL identified in this study were more refined with precision on the locations and flanking markers to facilitate their integration into breeding programs. Since the available algorithms did not allow a direct integration of association studies in the meta-analysis, we further superimposed physical positions of 178 GWAS-detected MTA with the MQTL intervals. Depending on the chromosome, about 7−50% of MTA from six independent studies fell within different MQTL (**Table 6**). This firstly shows the high quality of our MQTL analysis, and secondly suggests the need for new bioinformatic tools that can integrate association mapping studies in meta-analysis to better elucidate genetic basis of FER- and GER-related traits, and find interesting loci that might be included in trait introgression strategies. Furthermore, FER and GER resistance- and susceptibility-promoting genes, and underlying molecular mechanisms were also discussed within 14 most refined MQTL through a transcriptomic analysis using recently published RNA-Seq data by Kebede et al. (2018). We will include in the discussion also results from relevant papers that could not be included in the meta-analysis because they did not fulfil the basic requirements.

### Co-inheritance of Fusarium and Gibberella ear rot resistances in maize

Our results revealed that the most refined MQTL *ZmMQTL1.*5 (243.46−259.01 Mbp) and *ZmMQTL2.*2 (13.3−20.58 Mbp) with PVE>10% were specific to FER and GER, respectively (**Figure 2**, **Table 7**). This confirms that Fusarium and Gibberella ear rots are two different types of maize ear rots, and breeding for resistance to these diseases can be implemented separately. In contrast, 28 of the 40 MQTL identified in this study were common to both FER and GER resistances and were distributed across all chromosomes. This impressive number of common genomic loci offers a great opportunity to breed for multiple resistance to ear rots, particularly in maize production areas prone to both FER and GER. Previous meta-analysis by Xiang et al. (2010) also revealed 15 MQTL conferring resistance to both FER and GER. In addition, Giomi et al. (2016), also reported four QTL for both FER and GER using a multi-trait multiple interval mapping in an Argentinian mapping population. Furthermore, the relationship between FER and GER has been phenotypically investigated by Löffler et al. (2010a) who found flint and dent genotypes which were resistant to both diseases. Depending on the testing years, Schaafsma et al. (2006) found moderate to strong correlations (r = 0.40−0.75) between FER and GER resistances in different sets of Canadian commercial hybrid cultivars. Butrón et al. (2015) also reported a highly significant correlation (r = 0.71) between FER and GER resistances. These authors concluded that breeding for resistance to FER would more likely affect resistance to GER and vice versa. These findings emphasize that improving multiple resistance to FER and GER is feasible and can be efficiently achieved through the integration of identified common MQTL into breeding programs.

### Meta-QTL and types of ear rot resistance

For both FER and GER, the existence of specific MQTL for SR (e.g. *ZmMQTL3.1* and *ZmMQTL9.1*) and KR (e.g. *ZmMQTL1.5*, *ZmMQTL2.4*) (**Figures 2**, **3**, **Supplementary File 4**) demonstrates that silk and kernel resistances represent two major types of active resistance reactions to ear rot diseases in maize as previously reported by Reid et al. (1996a); Chungu et al. (1996); Plienegger and Lemmens (2002); Mesterházy et al. (2012) and Kebebe et al. (2015). Reinprecht et al. (2008) also demonstrated that silk and kernel resistances were two different traits to be considered when breeding for GER resistance in maize. The main difference between the two types resides in the inoculation techniques used, mimicking different pathogen entry modes (Chungu et al., 1996). Silk resistance occurs after inoculation of the silk channel, while kernel resistance occurs after inoculation in the middle of the ear. Under natural conditions, the fungus can enter the ear *via* the silk channel (silk resistance), and directly through wounds created by hail, insects or agricultural tools and machines (kernel resistance) (Nerbass et al., 2016; Blandino et al., 2017).

Our study identified four MQTL for both silk and kernel resistances of FER, and 15 MQTL for kernel resistance of FER and silk resistance of GER. Eleven MQTL were also found to control both silk and kernel resistances of GER (**Table 4**). This finding indicates the existence of genomic regions with multiple resistance which could be exploited in breeding programs aiming to improve ear rot resistance in maize. Based on SSR, RFLP and RADP markers, Ali et al. (2005) also reported one genomic region located on chromosome 1 (*BC373_650-S116_1*) and one on chromosome 7 (*BC324_1400-umc1407*) which controlled both silk and kernel GER resistances. In addition, the relationship between the two types of resistances was investigated by Chungu et al. (1996) who found positive strong phenotypic correlations (r = 0.77−0.89). Moderate correlation (r = 0.66) was reported between the two traits by Löffler et al. (2010b). Similarly, Kebebe et al. (2015) reported moderate to very strong genotypic correlations (r_g_ = 0.60−0.99) between the two traits and demonstrated that both silk channel and kernel inoculation techniques ranked genotypes in a similar way. From the 19 MQTL, eight were identified as the most refined MQTL explaining considerable phenotypic variance (average PVE = 10−17%) with 2−7 overlapping QTL which were identified from 2−5 populations evaluated across different environments (**Table 7**). This firstly exhibits these MQTL as important genomic loci controlling both types of resistance, and secondly implies that the integration of these MQTL into breeding programs is likely to improve stable multiple resistances to FER and GER due to both silk channel and kernel infections. Both resistance types are important for environments where the European corn borer (*Ostrinia nubilalis*) regularly occurs, because the insect-driven wounding of the cob in the 2^nd^ generation of the insect might result in strong kernel infection additionally to silk infection that mainly occurs when it rains during silking. With this, the use of insect resistant genotypes under natural conditions (and without any other wounding factors), would reduce fungal infection of the kernels even if the genotypes are not resistant to the fungi. This could lead to co-occurrence of resistance QTL for both diseases although they have genetically nothing in common. So far, co-localization of genomic regions for insect and fungal resistances has not been established for maize ear rots.

### Colocalization of genomic regions controlling KR, SR and mycotoxin accumulation

DON shared two MQTL with KR of FER and/or GER (*ZmMQTL1.1* and *ZmMQTL1.7*) and two MQTL with SR of FER and/or GER (*ZmMQTL1.1* and *ZmMQTL2.1*) (**Table 4**, **Supplementary File 4**). Similarly, FUM shared three MQTL with KR of FER (*ZmMQTL1.6*, *ZmMQTL3.7* and *ZmMQTL6.1*) and three with SR of FER and/or GER (*ZmMQTL4.1*, *ZmMQTL6.1* and *ZmMQTL7.3*). This indicates the existence of common genomic regions between mycotoxin accumulation and the two types of active resistance in maize. For GER, Martin et al. (2011) using SSR markers to analyze 150 DH lines derived from UH007×UH006, also found one QTL on chromosome 2 which was common to DON accumulation and silk resistance. This was supported by the existence of a strong positive genotypic correlation (r = 0.95) between the two traits (Martin et al., 2011). In addition, Szabo et al. (2018) detected strong positive correlations between GER severity and DON contamination with correlations of r = 0.95 and r = 0.82 for *F. graminearum* and *F. culmorum*, respectively. They concluded that GER resistance is an important indicator of lower toxin contamination. Genotypes with higher GER resistance would have lower DON accumulation as indicated by Bolduan et al. (2009). Similar observations were made by Miedaner et al. (2015) who found moderate to strong correlations (r = 0.60−0.90) between DON measured by immunotests and GER severity, indicating that DON could be predicated by GER severity. For FER, Butrón et al. (2015) observed strong correlations (r = 0.97) between disease severity and FUM. Similarly, Cao et al. (2022) found strong genotypic correlation (r_g_ = 0.85) between FUM and FER severity after kernel inoculation. Based on this, selection for FER-resistant lines would indirectly reduce fumonisins accumulation (Maschietto et al., 2017; Galić et al., 2019).

However, our analysis also revealed the existence of one specific QTL for FUM (*qFER12*, PVE = 8%) on chromosome 5 (**Figure 2**) and one for DON (*qGER12*, PVE = 15%) on chromosome 9 (**Figure 3**), which were identified as independent MQTL for these traits. This implies that it would be more relevant to consider evaluating DON and FUM as separate traits from FER and GER severity, particularly if the breeder targets those specific genomic regions. Although resistant genotypes had generally low toxin contamination, Reid et al. (1996b) and Dalla Lana et al. (2022) demonstrated that the relationship between DON and GER severity was more complex and non-linear. Genotypes with different disease severity might exhibit similar mycotoxin concentrations. In wheat, Wang et al. (2021) investigating the complex relationship between FHB and DON, found individual genotypes with low disease severity that exhibited high DON accumulation. In the USA, Dalla Lana et al. (2021) analyzed DON in maize ears over four years and showed that its accumulation was affected by multiple weather conditions. They indicated that from a total of 483 asymptomatic ears, 196 (about 41%) exhibited detectable level of 0.05 mg/kg for DON accumulation, and 46 (approximately 10%) showed 1−5 mg/kg of DON. Moreover, Mesterhazy et al. (2022) evaluated 18 commercial maize hybrids from Hungary for different ear rots including FER and GER, and observed a lack of phenotypic correlations between ear rot resistance and toxins, indicating that toxins analysis is necessary. Therefore, indirect selection for DON or FUM using disease severity would be feasible and more effective through the exploitation of identified common MQTL, however, advanced lines should be further analyzed for DON and/or FUM accumulation in a later stage of the selection cycle. Furthermore, MQTL *ZmMQTL9.4* (145.46−151.40 Mbp) on chromosome 9 was common to FUM and DON. This firstly demonstrates the existence of genomic regions with resistance to multiple mycotoxin accumulation, and secondly indicates that selection for resistance to one mycotoxin using this MQTL would reduce accumulation of the other mycotoxin. The same has been reported on the basis of phenotypic data by Miedaner et al. (2015) for the co-occurrence of resistances to DON and zearalenone, another mycotoxin produced by *F. graminearum*.

### Morphological traits and their association with FER and GER infections in maize

Several MQTL for SR and KR of both FER and GER were also detected in association with KDD (e.g. *ZmMQTL1.1* and *ZmMQTL6.1*), and HC (*ZmMQTL1.4*, and *ZmMQTL6.1*) (**Table 4**, **Supplementary File 4**). This indicates that morphological traits such as kernel dry-down rate and husk coverage may have a passive contribution to both silk and kernel resistances in maize. Kernel dry-down rate and husk coverage represent natural barriers which reduce infection by blocking the pathogen entry into the ear or the kernel. Passive resistance due to morphological traits was also reported for FHB disease in wheat by several studies (Mesterházy, 1995; Buerstmayr and Buerstmayr, 2015; Buerstmayr et al., 2020; Ruan et al., 2020; Xu et al., 2020). Husk characteristics were reported as important traits in protecting the ears from pathogen infection (Warfield and Davis, 1996; Jiang et al., 2020). Butoto et al. (2022) found a low negative correlation (r = −0.30) between husk coverage and FER severity. In addition, moderate genotypic correlations (r = 0.39−0.61) were detected between husk coverage and Diplodia ear rot severity due to *Stenocarpella maydis* infection across three locations (Rossouw et al., 2002). The positivity of the correlations found by Rossouw et al. (2002) is explained by the fact that the authors evaluated the husk coverage based on a scale opposite to the previous paper. Therefore, the tighter the husk over the ear, the lower the ear rot severity.

Common genomic regions were also reported by Xiang et al. (2012) when investigating the relationships between grain moisture content and ear rot resistance in maize. Depending on the maturity stage of the kernels, Kebebe et al. (2015) found in Canada moderate to strong negative genotypic correlations between kernel dry-down rate and silk resistance (r = −0.58 to −0.90) and kernel resistance (r = −0.67 to −0.79) for GER. Thus, genotypes with fast drying kernels would have relatively lower GER severity. Substantially high selection efficiencies (0.52−0.84) were observed by Kebebe et al. (2015) when selecting for less kernel infection using kernel dry-down rate, whereas lower selection efficiencies (0.29−0.32) were found for silk channel infection. Since silk inoculation is usually earlier (5-6 days post silking) than kernel inoculation (15-21 days post silking), the infection through silk channel would have significantly progressed before the onset of kernel dry-down. This indicates that despite the existence of common genomic loci between kernel dry-down rate and FER and GER resistances, the use of kernel dry-down rate as an indirect trait to improve ear rot resistance might not be as effective as the direct selection for disease severity, especially for SR. Moreover, additional investigations are required to elucidate the interactions between kernel dry-down rate and grain yield and related traits in maize.

### Resistance and susceptibility genes controlling FER and GER in maize

Based on transcriptomic data reported by Kebede et al. (2018) for GER, 59 candidate genes harbored by 14 of the MQTL identified in this study were differentially expressed in one resistant line (CO441) and one susceptible line (B37) after inoculation with *F. graminearum* (**Supplementary File 7**). This emphasizes the importance of these MQTL as targets for improving multiple resistance to ear rot diseases in maize. Thirteen of these candidate genes were annotated as “*uncharacterized protein*” (**Supplementary File 8**), and therefore require further investigations to characterize corresponding proteins to better elucidate their roles in the resistance or susceptibility to ear rot in maize. GER-specific MQTL *ZmMQTL2.2* and the common MQTL *ZmMQTL9.4* harbored two different defense response genes such as *GRMZM2G342033* and *GRMZM2G423331*, respectively. Similarly, the common MQTL *ZmMQTL9.2* (113.95−129.03 Mbp) harbored two defense response genes, namely *GRMZM2G011151* and *GRMZM2G093092* which were specific to CO441. In comparison to the susceptible line, the expression levels of *GRMZM2G342033* and *GRMZM2G423331* at 2 DAI in CO441 were constitutively stronger with TPM two-fold higher than that in B37.

*GRMZM2G342033* encoded “*S-norcoclaurine synthase2*” which had about 71.3% of identity with “*S-norcoclaurine synthase”* previously reported as a member of the pathogenesis-related protein 10 (PR10) family (Lee and Facchini, 2010; Nida et al., 2021). The PR10 family proteins have been extensively reported for their antifungal activity (Xie et al., 2010; Wu et al., 2016), and their crucial role in resistance against GER pathogens (Mohammadi et al., 2011). Xie et al. (2010) identified another PR10 gene (*ZmPR10.1*) on chromosome 10 which conferred resistance to Aspergillus ear rot caused by *Aspergillus flavus* in maize. Similarly, in a previous transcriptional analysis, Lanubile et al. (2014) also identified *GRMZM2G342033* as “*S-norcoclaurine synthase-like*” which was involved in resistance to FER in maize.

*GRMZM2G011151* was annotated as “*terpene synthase21* (*tps21*)*”* which has been previously reported by Ding et al. (2017) as a α/β-costic acid pathway candidate gene in maize. *tps21* enables the biosynthesis of α/β-selinene volatiles which are in turn converted into α/β-costic acids, promoting resistance to fungal pathogen infections (Block et al., 2019). α/β-costic acids are non-volatile diterpenoids which were demonstrated to inhibit growth of several fungal species including *F. graminearum, F. verticillioides*, *Rhizopus microsporus*, *Aspergillus parasiticus*, and *Cochliobolus heterostrophus* (Ding et al., 2017). Moreover, near-isogenic lines (NILs) lacking functional copies of *tps21* exhibited a high susceptibility to Fusarium species compared to functional NILs (Ding et al., 2017). Lanubile et al. (2014) also identified *GRMZM2G011151* as a defense response gene to FER which was specifically differentially expressed in CO441 compared to another susceptible line (CO354).

Similar to *GRMZM2G011151*, *GRMZM2G093092* and *GRMZM2G423331* were reported as candidate defense response genes to GER (Kebede et al., 2018), which encoded the “*flavonoid O-methyltransferase2 (fomt2)*” and “*flavonoid O-methyltransferase4 (fomt4)*” proteins, respectively. *FOMT2* and *FOMT4* proteins catalyze the biosynthesis of sakuranetin, a well-characterized flavonoid which negatively affected the germination of fungal spores in rice (Kodama et al., 1992; Hasegawa et al., 2014). *GRMZM2G423331* was also identified in a recent transcriptomic analysis by Förster et al. (2022) as a *FOMT4* gene which is involved in the flavonoid pathway related to a general response to *F. graminearum* and *F. verticillioides* in maize. Recently, Maschietto et al. (2017) found that *GRMZM2G093092* was uniquely expressed in CO441 compared to CO354 after infection with *F. verticillioides*. In addition, *FOMT2* and *FOMT4* enable cell-wall reinforcement and higher lignification which both inhibit fungus growth and the development of the disease. These results suggest the biosynthesis of different secondary metabolites or phytoalexins (e.g. terpenoid and flavonoid) which occurs after initial infection with FER- and GER-causing species. Moreover, Balcerzak et al. (2012) indicated that during the infection, fungus-specific genes like *feruloyl esterase (FAE)* are activated to enable the biosynthesis of pathogen-associated molecule patterns (PAMPs), like oligogalacturonides. These molecules firstly degrade the cell wall to facilitate the infection, and secondly are perceived as elicitors by pathogen recognition receptor kinases. This results in successive oxidation-reduction reactions leading to reaction oxygen species (ROS) production (Kebede et al., 2018; Yuan et al., 2020b) and the activation of defense response and phytoalexin-coding genes (Förster et al., 2022). Given the specificity of genes *GRMZM2G011151* and *GRMZM2G093092* to the resistant genotype, and the fact that they were harbored by a common MQTL (*ZmMQTL9.2*) to FER and GER, their incorporation into breeding programs would efficiently improve a broad-based resistance to both Fusarium and Gibberella ear rots in maize.

Furthermore, we also identified 36 candidate genes which were uniquely differentially expressed in the susceptible line, suggesting the existence of ear rot susceptibility genes in maize. The gene *GRMZM2G164405* harbored by *ZmMQTL2.2* encoded the “*1-aminocyclopropane-1-carboxylate synthase2 (acs2)*” protein which was involved in the biosynthesis of ethylene and pyridoxal phosphate binding activity. Since *ZmMQTL2.2* is a GER-specific MQTL, this finding demonstrates that ethylene-signaling pathway is associated with susceptibility to GER in maize as previously indicated by Kebede et al. (2018). Similar results were reported by Chen et al. (2009) who found that ethylene-signaling increased susceptibility and premature cell death after inoculation with *F. graminearum* and DON in wheat and barley (*Hordeum vulgare* L.). However, under infection with *F. verticillioides*, Maschietto et al. (2017) found that the expression level of gene *GRMZM2G053503* located on chromosome 8 at position 35.56 Mbp, was 1.23-fold higher in CO441 than in CO354. This gene encodes “*ethylene-responsive factor-like protein 1*” which is involved in resistance to FER in maize. Interestingly, *GRMZM2G053503* is located within the FER-specific MQTL *ZmMQTL8.2* (20.8−81.7 Mbp) which was not considered in our transcriptomic analysis. This demonstrates that the ethylene-signaling pathway plays differential roles in maize ear rot depending on the Fusarium species. In addition to *GRMZM2G164405*, another interesting susceptibility gene was *GRMZM2G146108* located within the MQTL *ZmMQTL9.4*. This gene was annotated as “*hemoglobin1 (hb1)*” which enabled programmed cell death in the susceptible line. So far, to the best of our knowledge, *GRMZM2G146108* has not been attributed to FER and/or GER susceptibility in maize, and thus merits further examination. The attenuation of the ethylene-signaling pathway could improve GER resistance in moderately to highly susceptible genotypes. This could be done through the application of RNA interference (RNA_i_) technology (Das and Sherif, 2020) on *GRMZM2G164405* as described for “*Ethylene Insensitive 2 (EIN2)*” gene with FHB and DON accumulation caused by *F. graminearum* in wheat and barley (Chen et al., 2009). Alternatively, the susceptibility genes could be knocked out by the clustered regularly interspaced short palindromic repeats (CRISPR) technology (Campenhout et al., 2019; Wada et al., 2020). Both attempts would also biologically validate the contribution of these genes in the maize/ear rot pathosystems.

### Strategies for the successful introgression of resistance genes to FER and GER into elite materials

Genetic resources from diverse geographical origins contributed to the 40 MQTL identified in this study (**Table 2**, **Tables 5**, **6**). In Europe, flint and dent germplasms including the “Kemater Landmais Gelb” (KE) landrace population harbored several resistance alleles which could be introgressed into elite cultivars for enhanced ear rot resistance (Han et al., 2016; Han et al., 2018; Gaikpa et al., 2021). However, FER and GER resistances are complex polygenic traits, and our results demonstrated that more than 65% of the MQTL had minor (PVE<10%) effects on the respective traits. This indicates that the exploitation of these MQTL using marker-assisted selection (MAS) would require intensive breeding and marker efforts and might not yield a significant selection gain. Although MAS has been successfully implemented to improve traits controlled by one or a few large-effect genes in several crops (Kuchel et al., 2007; Hasan et al., 2021), its potential in improving complex traits remains limited as previously discussed in wheat and barley by Miedaner and Korzun (2012). As implication, the successful introgression of the resistance genes for stronger and durable multi-disease resistances, calls for more advanced and sophisticated genomic approaches, like genomic selection (Bhat et al., 2016; Gaikpa and Miedaner, 2019; Budhlakoti et al., 2022). For FER and GER resistances, this could be achieved through the application of the integrated genomics-assisted breeding scheme suggested by Miedaner et al. (2020). This approach is implemented in two steps, including: (i) introgression of the resistant donor (e.g. KE lines) by backcrossing to the susceptible line used as recurrent parent without marker selection, and (ii) application of genomic selection following a recurrent selection scheme for an accelerated selection for FER and/or GER resistances as well as adaptation traits (Miedaner et al., 2020). Identified MQTL can be efficiently incorporated in the genomic selection model built in the second step.

## Conclusions

Understanding the genetic basis and molecular mechanisms controlling Fusarium and Gibberella ear rots is a key requirement for the development of maize varieties with improved multi-disease resistances and related traits. Based on 164 projected QTL from 15 studies, we demonstrated the existence of 40 MQTL which revealed colocalization of genomic regions governing FER and GER silk and kernel resistances, FUM and DON accumulation, kernel dry-down rate and husk coverage. Three of the most refined MQTL (*ZmMQTL2.2*, *ZmMQTL9.2* and *ZmMQTL9.4*) for FER- and/or GER-related traits harbored promising resistance genes which were constitutively and strongly expressed in the resistant line (CO441) analyzed in the published transcriptomic study by Kebede et al. (2018). The effectiveness of the introgression of these candidate genes from identified sources of resistance into susceptible varieties through genomics-assisted backcross breeding strategies need to be explored to systematically improve ear rot resistances while reducing mycotoxins contamination in maize.

## Data availability statement

The original contributions presented in the study are included in the article/**Supplementary Material**. Further inquiries can be directed to the corresponding author.

## Author contributions

FA designed the study, conducted literature search, conducted data analysis and wrote the manuscript. TM conducted an in-depth review of the manuscript. All authors contributed to the article and approved the submitted version.

## Funding

This research was partly funded by the German Academic Exchange Service, Bonn, Germany as a doctoral study grant to Félicien Akohoue (grant no. 91770158) and partly by the University of Hohenheim.

## Acknowledgments

We wish to express our sincere gratitude to Mr. Serge Alindekon, University of Rostock, Germany, for his valuable help with literature search using Web of Science. Many thanks to Prof. Linda J. Harris from Eastern Cereal and Oilseed Research Centre, Agriculture and Agri-Food Canada, Ottawa, Canada, and Prof. Guangfei Zhou from State Key Laboratory for Crop Genetics and Germplasm Enhancement, Nanjing Agricultural University, China, for sharing with us genetic maps used in their respective studies.

## Conflict of interest

The authors declare that the research was conducted in the absence of any commercial or financial relationships that could be construed as a potential conflict of interest.

## Publisher’s note

All claims expressed in this article are solely those of the authors and do not necessarily represent those of their affiliated organizations, or those of the publisher, the editors and the reviewers. Any product that may be evaluated in this article, or claim that may be made by its manufacturer, is not guaranteed or endorsed by the publisher.
