# Supplementary material for: Meta-analysis and co-expression analysis revealed stable QTL and candidate genes conferring resistances to Fusarium and Gibberella ear rots while reducing mycotoxin contamination in maize
Source: Front Plant Sci. 2022 Oct 31;13:1050891. doi: 10.3389/fpls.2022.1050891 (PMC9662303; doi:10.3389/fpls.2022.1050891)
Supplement: Supplementary file 1 [file DataSheet_1.zip › Supplementary Files 1-8/Supplementary File 2.pdf]

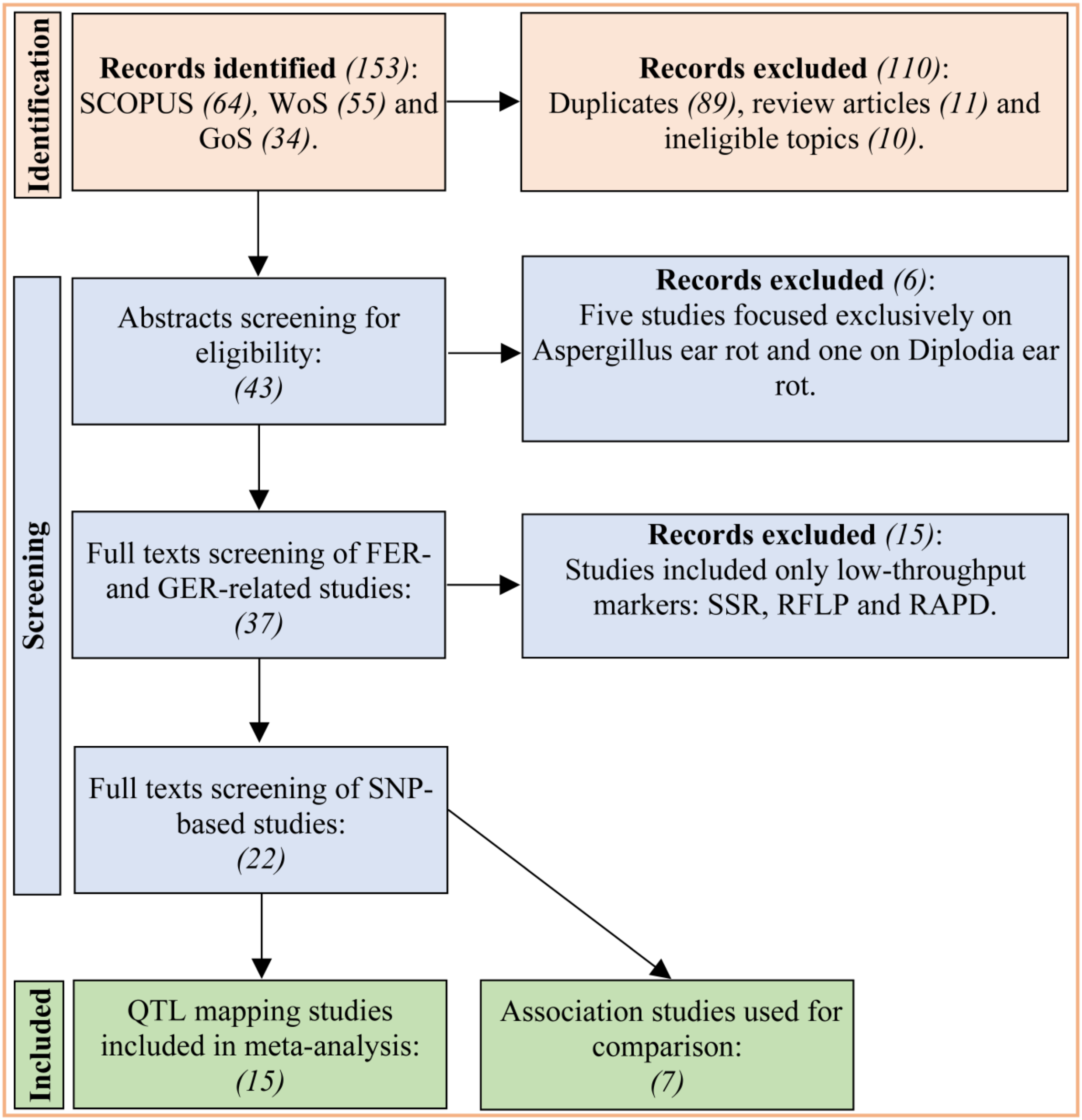

Supplementary File 2: Preferred reporting items for systematic review and meta-analyses (PRISMA) flow diagram. WoS = Web of Science, GoS = Google Scholar, RAPD = random amplified polymorphic DNA, RFLP = restriction fragment length polymorphisms, SNP = single nucleotide polymorphism, SSR = single sequence repeats. Numbers of publications are indicated in parenthesis for each category
