# Supplementary material for: Meta-analysis and co-expression analysis revealed stable QTL and candidate genes conferring resistances to Fusarium and Gibberella ear rots while reducing mycotoxin contamination in maize
Source: Front Plant Sci. 2022 Oct 31;13:1050891. doi: 10.3389/fpls.2022.1050891 (PMC9662303; doi:10.3389/fpls.2022.1050891)
Supplement: Supplementary file 1 [file DataSheet_1.zip › Supplementary Files 1-8/Supplementary File 5.pdf]

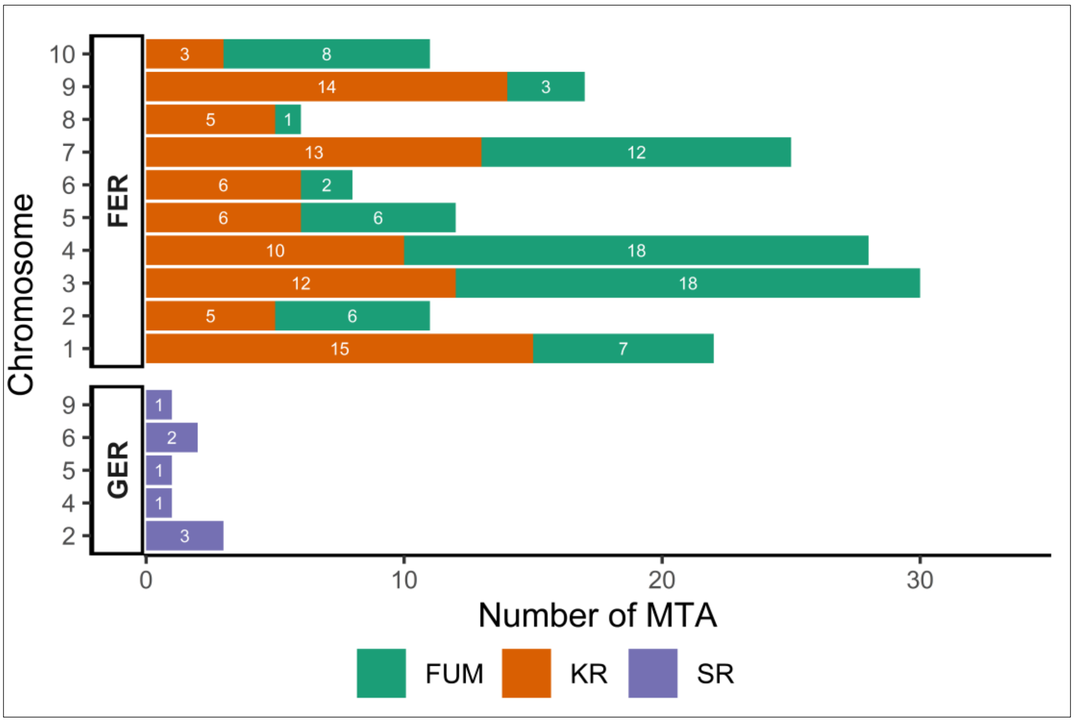

Supplementary File 5: Marker-trait associations (MTA) from genome-wide association study (GWAS) reported for Fusarium ear rot (FER) and Gibberella ear rot (GER). FUM = fumonisin accumulation, KR = kernel resistance, SR = silk resistance
